# Supplementary material for: Arbuscular Mycorrhizal Fungi Improve the Growth, Water Status, and Nutrient Uptake of Cinnamomum migao and the Soil Nutrient Stoichiometry under Drought Stress and Recovery
Source: J Fungi (Basel). 2023 Mar 5;9(3):321. doi: 10.3390/jof9030321 (PMC10057927; doi:10.3390/jof9030321)
Supplement: Supplementary file 1 [file jof-09-00321-s001.zip › jof-2187413-supplementary.pdf]

Arbuscular Mycorrhizal Fungi improve the growth, water status, and nutrient uptake of *Cinnamomum migao* and the soil nutrient stoichiometry under drought stress and recovery

Supplementary Materials:

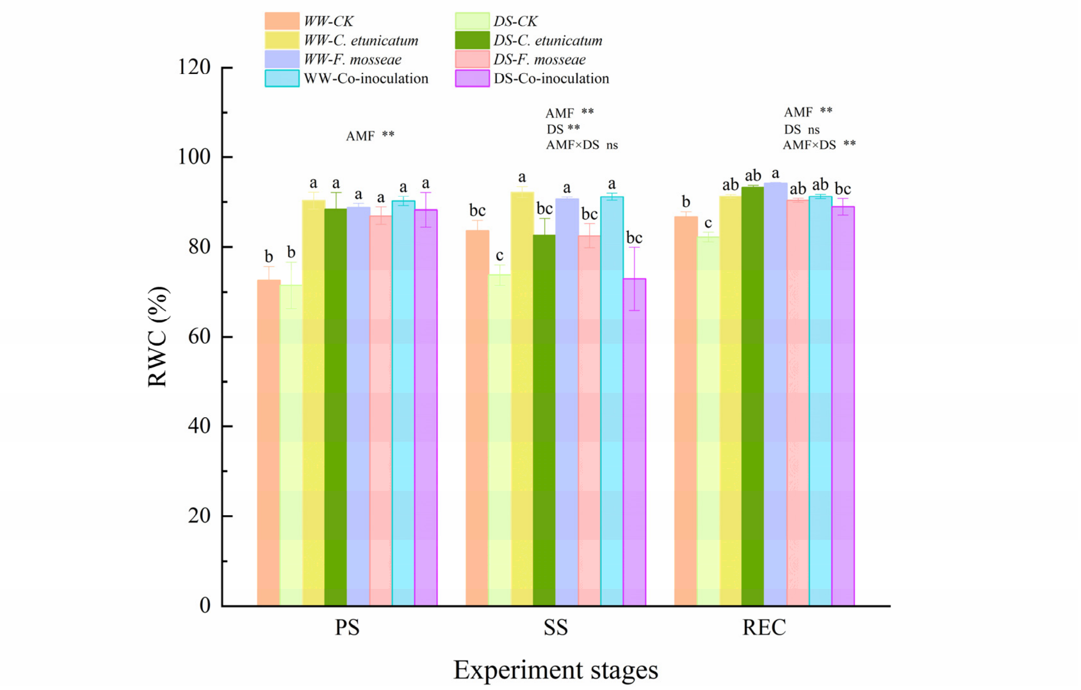

Figure S1. Effect of arbuscular mycorrhizal fungus (AMF) on the RWC of *C. migao* seedlings measured at different experiment stages. Different letters (a, b, c, d) indicate a significant difference of Tukey's post hoc test (** $p < 0.01$; ns, not significant) between all treatments. PS: prior stress, SS: subjected to drought stress; REC: rewatered; DS: drought stress; WW: well-watered. CK, non-AMF control; *C.etunicatum*, plants inoculated with *C.etunicatum*; *F.mosseae*, plants inoculated with *F.mosseae*; Co-inoculation, plants inoculated with *F.mosseae* & *C.etunicatum*. Values are expressed as the mean ± SE (n = 6, which are treatment replicates).

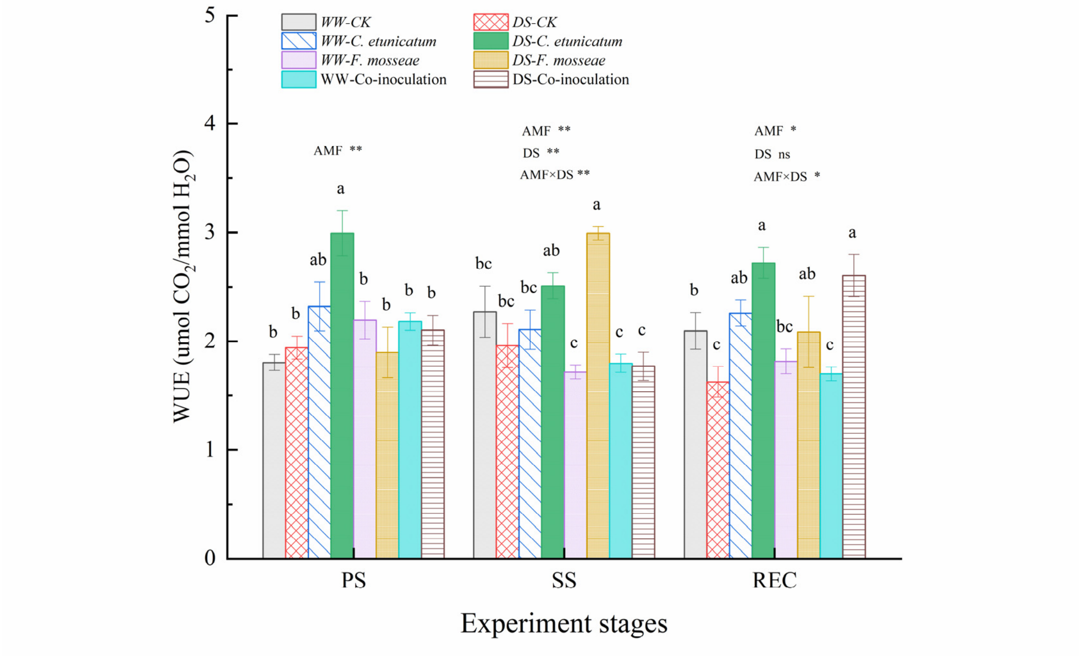

Figure S2. Effect of arbuscular mycorrhizal fungus (AMF) on the WUE of *C. migao* seedlings measured at different experiment stages. Different letters (a, b, c, d) indicate a significant difference of Tukey's post hoc test (** $p < 0.01$; * $p< 0.05$; ns, not significant) between all treatments. PS: prior stress, SS: subjected to drought stress; REC: rewatered; DS: drought stress; WW: well-watered. CK, non-AMF control; *C.etunicatum*, plants inoculated with *C.etunicatum*; *F.mosseae*, plants inoculated with *F.mosseae*; Co-inoculation, plants inoculated with *F.mosseae* & *C.etunicatum*. . Values are expressed as the mean ± SE (n = 6, which are treatment replicates).

Table S1. Effect of arbuscular mycorrhizal fungus (AMF) on the Leaf dry mass, Stem dry mass, and Root dry mass of *C. migao* seedlings measured at different experiment stages. Different letters (a, b, c, d) indicate a significant difference of Tukey's post hoc test (** p < 0.01; * p< 0.05; ns, not significant) between all treatments. PS: prior stress, SS: subjected to drought stress; REC: rewatered; DS: drought stress; WW: well-watered. CK, non-AMF control; *C.etunicatum*, plants inoculated with *C.etunicatum*; *F.mosseae*, plants inoculated with *F.mosseae*; Co-inoculation, plants inoculated with *F.mosseae* & *C.etunicatum*. Values are expressed as the mean ± SE (n = 6, which are treatment replicates).

water regimes	AMF status	Leaf dry mass (g)			Stem dry mass (g)			Root dry mass		
		PS	SS	REC	PS	SS	REC	PS	SS	REC
DS	CK	7.65±0.52d	10.58±0.97d	11.65±0.96d	5.49±0.65b	9.30±2.02c	11.76±2.04d	4.96±0.66c	7.15±0.85c	8.06±0.74e
	C. etunicatum	22.64±1.57a	23.39±2.08ab	25.94±2.07ab	15.12±1.11a	19.90±1.28a	21.72±1.80ab	9.52±0.77a	11.23±0.89ab	12.13±1.27bcd
	F. mosseae	16.75±1.03bc	18.37±1.19bc	20.67±1.23bc	11.89±0.52a	16.48±0.57ab	19.03±0.95bc	8.74±1.01ab	11.00±1.16abc	12.19±1.10bc
	Co-	13.85±1.39c	14.28±1.71cd	15.24±1.84cd	12.40±1.01a	16.66±0.99ab	18.07±0.64bc	6.87±0.95bc	8.27±0.73bc	9.16±0.49cde
WW	CK	6.67±0.82d	12.18±1.07cd	15.42±1.80cd	5.07±0.75b	12.19±0.79bc	13.96±1.07cd	4.49±0.66c	7.51±0.81bc	8.10±0.86de
	C. etunicatum	23.05±1.64a	26.89±2.31a	29.67±2.58a	15.57±1.34a	22.08±1.59a	25.98±0.58a	10.06±0.91a	13.10±0.96a	16.60±0.24a
	F. mosseae	17.81±1.05b	22.94±0.62ab	26.15±0.83ab	12.11±0.51a	20.82±0.80a	22.90±1.29ab	9.17±0.65ab	12.54±0.95a	13.38±1.28ab
	Co-	14.29±1.45bc	17.31±1.19bcd	19.44±0.72bc	13.17±1.09a	19.64±1.10a	22.99±1.18ab	7.97±0.73ab	11.05±0.58abc	12.83±0.43abc
Significance										
AMF		**	**	**	**	**	**	**	**	**
DS		ns	**	**	ns	**	**	ns	*	**
AMF×DS		ns	ns	ns	ns	ns	ns	ns	ns	ns

Table S2. Effect of arbuscular mycorrhizal fungus (AMF) on the total concentration of carbon in leaves (leaf SOM), stems (stem SOM) and roots (root SOM) of *C. migao* seedlings measured at different experiment stages. Different letters (a, b, c, d) indicate a significant difference of Tukey's post hoc test (** $p < 0.01$; * $p< 0.05$; ns, not significant) between all treatments. PS: prior stress, SS: subjected to drought stress; REC: rewatered; DS: drought stress; WW: well-watered. CK, non-AMF control; *C.etunicatum*, plants inoculated with *C.etunicatum*; *F.mosseae*, plants inoculated with *F.mosseae*; Co-inoculation, plants inoculated with *F.mosseae* & *C.etunicatum*. Values are expressed as the mean ± SE (n = 6, which are treatment replicates).

water regimes	AMF status	Leaf SOM (g/kg)			Stem SOM (g/kg)			Root SOM(g/kg)		
		PS	SS	REC	PS	SS	REC	PS	SS	REC
DS	CK	267.74±6.21c	242.43±4.89d	235.83±2.46d	234.58±4.14	225.47±2.70a	231.92±6.60a	168.90±6.31a	125.85±8.64d	153.21±15.32a
	C.	302.58±2.54ab	275.64±6.89bc	283.98±5.47bc	80.03±1.46c	103.89±3.39c	168.00±34.33	189.19±4.46a	132.23±12.86cd	156.48±9.95ab
	F. mosseae	282.69±4.92abc	256.94±5.24cd	264.86±4.72c	81.73±7.30c	104.37±6.40c	193.13±20.05	170.45±12.25	139.37±10.82bc	152.32±7.18b
	Co-	274.75±15.27ab	255.74±9.73cd	266.00±9.80c	163.71±5.28	114.74±3.84c	175.42±16.90	173.44±5.96a	129.98±7.70cd	165.53±12.88a
WW	CK	266.38±5.78c	275.23±6.53bc	283.67±6.92bc	221.53±3.51	237.28±2.73a	252.61±10.32	167.61±10.88	176.89±10.46ab	187.17±9.56a
	C.	304.85±1.69a	307.95±0.88a	309.17±1.52a	80.66±0.81c	89.84±3.91d	102.14±7.19c	190.94±2.90a	185.52±3.66a	182.23±3.17ab
	F. mosseae	285.94±4.00bc	288.11±1.62ab	285.79±3.07ab	81.29±6.48c	91.63±3.06d	116.24±7.74c	172.44±9.75a	169.02±8.04abc	178.53±6.12ab
	Co-	285.90±11.51bc	295.09±10.26a	298.05±1.65ab	165.33±6.48	159.91±5.21b	168.01±6.71b	167.32±3.16a	166.48±2.95abc	165.53±12.88a
Significanc										
AMF		**	**	**	**	**	**	*	ns	ns
DS		ns	**	**	ns	*	**	ns	**	*
AMF×DS		ns	ns	ns	ns	**	**	ns	ns	ns

Table S3. Effect of arbuscular mycorrhizal fungus (AMF) on the total concentration of nitrogen in leaves (leaf TN), stems (stem TN) and roots (root TN) of *C. migao* seedlings measured at different experiment stages. Different letters (a, b, c, d) indicate a significant difference of Tukey's post hoc test (** $p < 0.01$; * $p < 0.05$; ns, not significant) between all treatments. PS: prior stress, SS: subjected to drought stress; REC: rewatered; DS: drought stress; WW: well-watered. CK, non-AMF control; *C.etunicatum*, plants inoculated with *C.etunicatum*; *F.mosseae*, plants inoculated with *F.mosseae*; Co-inoculation, plants inoculated with *F.mosseae* & *C.etunicatum*. Values are expressed as the mean ± SE (n = 6, which are treatment replicates).

water regimes	AMF status	Leaf TN (g/kg)			Stem TN (g/kg)			Root TN (g/kg)		
		PS	SS	REC	PS	SS	REC	PS	SS	REC
DS	CK	194.84 ±	156.77±6.75d	150.26±7.20d	53.97±	33.46±	29.06±	92.47±5.38cd	101.21±3.01d	113.96±
	C. etunicatum	198.38 ±	186.00±4.97c	191.56±3.54bc	29.55±	27.18±	23.67±1.22c	129.35±7.20a	145.24±7.32a	150.59±5.14a
	F. mosseae	188.11±4.13c	151.48±9.73d	160.28±	28.73±	25.89±	22.33±0.52c	108.43±	123.79±	126.57±
	Co-	235.49±3.66a	185.43 ±	194.71±	29.43±	29.02±	22.38±0.33c	126.20±	133.38±	138.47±4.23ab
WW	CK	191.81 ±	200.79±2.75b	206.64±15.00b	49.78±	50.69±	48.97±4.66a	85.19±3.59d	96.47±8.95d	103.60±8.58e
	C. etunicatum	206.88±2.78b	210.75±2.47b	217.39±15.00b	31.17±	31.93±	32.30±0.41b	130.46±0.53a	132.80±	133.43±
	F. mosseae	193.54 ±	151.48±9.73b	210.28±15.00b	28.73±	31.44±	31.26±0.62b	109.69±	110.73±	109.59±1.34de
	Co-	240.49±2.78a	230.55±2.17a	252.33±15.00a	33.11±	31.96±	33.80±0.50b	127.85±2.16a	124.70±	128.98±
Significance										
AMF		**	**	**	**	**	ns	**	**	**
DS		ns	**	**	ns	**	**	ns	**	**
AMF×DS		ns	*	*	ns	ns	*	ns	ns	ns

Table S4. Effect of arbuscular mycorrhizal fungus (AMF) on the total concentration of phosphorus in leaves (leaf TP), stems (stem TP) and roots (root TP) of *C. migao* seedlings measured at different experiment stages. Different letters (a, b, c, d) indicate a significant difference of Tukey's post hoc test (** $p < 0.01$; * $p < 0.05$; ns, not significant) between all treatments. PS: prior stress, SS: subjected to drought stress; REC: rewatered; DS: drought stress; WW: well-watered. CK, non-AMF control; *C.etunicatum*, plants inoculated with *C.etunicatum*; *F.mosseae*, plants inoculated with *F.mosseae*; Co-inoculation, plants inoculated with *F.mosseae* & *C.etunicatum*. Values are expressed as the mean ± SE (n = 6, which are treatment replicates).

water regimes	AMF status	Leaf TP (g/kg)			Stem TP (g/kg)			Root TP (g/kg)		
		PS	SS	REC	PS	SS	REC	PS	SS	REC
WW	CK	0.93±0.02b	0.79±0.03e	0.63±0.03c	0.45±0.03c	0.24±0.02f	0.21±0.01f	0.46±0.01d	0.66±0.03e	0.70±0.03e
	C. etunicatum	1.81±0.09a	1.35±0.11bc	1.18±0.16b	1.45±0.05a	1.24±0.03b	1.28±0.02bc	2.27±0.05a	2.51±0.05a	2.65±0.05a
	F. mosseae	1.57±0.03a	1.09±0.06cd	1.19±0.09b	1.41±0.12a	1.02±0.02c	1.16±0.05cd	1.84±0.16b	1.86±0.11c	1.94±0.13c
	Co-	1.48±0.16a	1.24±0.07cd	0.73±0.10c	0.97±0.02b	0.76±0.02d	1.31±0.04b	1.98±0.08ab	2.06±0.10bc	2.14±0.09bc
DS	CK	0.90±0.02b	1.00±0.07de	1.19±0.09b	0.45±0.02c	0.51±0.06e	0.57±0.02e	0.43±0.02d	0.46±0.02e	0.46±0.02e
	C. etunicatum	1.87±0.08a	1.71±0.07a	1.74±0.02a	1.58±0.06a	1.54±0.04a	1.52±0.03a	2.30±0.04a	2.33±0.04ab	2.30±0.02b
	F. mosseae	1.61±0.02a	1.59±0.02ab	1.53±0.03ab	1.56±0.03a	1.35±0.03b	1.19±0.01bcd	1.33±0.04c	1.32±0.02d	1.36±0.03d
	Co-	1.51±0.16a	1.35±0.06bc	1.40±0.06ab	1.02±0.02b	1.04±0.02c	1.09±0.01d	1.94±0.03b	1.96±0.03c	1.97±0.03c
Significance										
AMF		**	**	**	**	**	**	**	**	**
DS		ns	**	**	*	**	**	*	**	**
AMF×DS		ns	*	ns	ns	ns	**	**	**	**

Table S5. Effect of arbuscular mycorrhizal fungus (AMF) on carbon: nitrogen in leaves (leaf C:N), stems (stem C:N) and roots (root C:N) of *C. migao* seedlings measured at different experiment stages. Different letters (a, b, c, d) indicate a significant difference of Tukey's post hoc test (** $p < 0.01$; * $p < 0.05$; ns, not significant) between all treatments. PS: prior stress, SS: subjected to drought stress; REC: rewatered; DS: drought stress; WW: well-watered. CK, non-AMF control; *C.etunicatum*, plants inoculated with *C.etunicatum*; *F.mosseae*, plants inoculated with *F.mosseae*; Co-inoculation, plants inoculated with *F.mosseae* & *C.etunicatum*. Values are expressed as the mean ± SE (n = 6, which are treatment replicates).

water regimes	AMF status	Leaf C:N			Stem C:N			Root C:N		
		PS	SS	REC	PS	SS	REC	PS	SS	REC
DS	CK	1.38 ± [illegible]	1.55 ± [illegible]	1.58±0.07abc	4.58±0.51a	6.87±0.52a	8.26±0.78a	1.86± [illegible]	1.25± [illegible]	1.35±0.13bc
	C. etunicatum	1.53±0.04a	1.49 ± [illegible]	1.48±0.04bcd	2.74±0.15b	3.86±0.20c	7.19±0.82ab	1.48±0.10c	0.90±0.05b	1.04±0.05c
	F. mosseae	1.51±0.04a	1.72 ± [illegible]	1.68±0.11ab	2.90±0.36b	4.20±0.62c	8.66±0.42a	1.57± [illegible]	1.13± [illegible]	1.20±0.06bc
	Co-[illegible]	1.17±0.08c	1.42 ± [illegible]	1.40±0.14cd	5.63±0.37a	6.02±0.33b	7.83±0.24ab	1.38±0.07c	0.97± [illegible]	1.19±0.07bc
WW	CK	1.39 ± [illegible]	1.37 ± [illegible]	1.38±0.05cd	4.71±0.50a	5.04± [illegible]	9.56±1.41a	1.99±0.17a	1.90±0.22a	1.86±0.20a
	C. etunicatum	1.48±0.02a	1.46 ± [illegible]	1.42±0.02bcd	2.59±0.07b	2.81±0.12d	3.17±0.12c	1.46±0.02c	1.40± [illegible]	1.37±0.03bc
	F. mosseae	1.48±0.04a	1.43±0.12a	1.36±0.12a	2.88±0.34b	2.92±0.09d	3.73±0.17c	1.57± [illegible]	1.53± [illegible]	1.63±0.05ab
	Co-[illegible]	1.19 ± [illegible]	1.19±0.05c	1.18±0.03d	5.01±0.19a	5.00± [illegible]	4.98±0.14bc	1.31±0.04c	1.33± [illegible]	1.28±0.10bc
Significance										
AMF		**	**	**	**	**	**	**	**	**
DS		ns	ns	ns	ns	**	**	ns	**	**
AMF×DS		ns	ns	ns	ns	**	**	ns	**	ns

Table S6. Effect of arbuscular mycorrhizal fungus (AMF) on carbon: phosphorus in leaves (leaf C:P), stems (stem C:P) and roots (root C:P) of *C. migao* seedlings measured at different experiment stages. Different letters (a, b, c, d) indicate a significant difference of Tukey's post hoc test (** $p < 0.01$; * $p < 0.05$; ns, not significant) between all treatments. PS: prior stress, SS: subjected to drought stress; REC: rewatered; DS: drought stress; WW: well-watered. CK, non-AMF control; *C.etunicatum*, plants inoculated with *C.etunicatum*; *F.mosseae*, plants inoculated with *F.mosseae*; Co-inoculation, plants inoculated with *F.mosseae* & *C.etunicatum*. Values are expressed as the mean ± SE (n = 6, which are treatment replicates).

water regimes	AMF status	Leaf C:P			Stem C:P			Root C:P		
		PS	SS	REC	PS	SS	REC	PS	SS	REC
DS	CK	287.16±5.83a	308.53±12.8a	378.27±19.17a	537.01±45.78	791.37±21.74	817.3±62.77a	367.81±23.57	238.43±21.11	240.55±13.84
	C.	169.28±8.64b	210.47±20.28c	262.55±39.07b	55.32±1.66c	84.03±3.42d	131.87±13.46c	83.56±3.07c	52.62±5.03c	58.92±3.3c
	F. mosseae	180.38±1.65b	238.87±13.76b	227.37±19.07c	59.86±7.06c	102.42±6.1cd	166.8±6.47c	94.03±6.65c	74.97±4.3bc	79.15±3.27bc
	Co-	191.03±16.42	208.56±14.14c	402.96±66.53a	168.71±8.13b	150.96±7.4c	134.69±8.69cd	88.2±5.04c	64.12±6.08c	78.59±8.24bc
WW	CK	297.16±14.04	281.74±29.42a	245.91±25.5bc	500.26±29.05	437.14±27.54	447.15±21.84b	395.64±16.35	273.58±25.44	333.07±43.29
	C.	162.56±7.02b	180.74±6.69d	177.46±1.68c	51.32±1.72c	58.19±1.24d	67.29±2.34d	83.25±2.11c	79.75±2.62bc	79.4±1.42bc
	F. mosseae	177.60±4.72b	181.41±2.55d	187.04±4.49c	52.22±4.21c	67.65±1.47d	97.57±2.83cd	129.26±6.62b	128.18±6.51b	131.2±3.56b
	Co-	194.62±15.29	220.35±10.71c	213.93±8.7c	162.04±5.21b	154.78±7.13c	154.02±2.9c	86.14±1.14c	85.15±2.24bc	84.24±6.43bc
Significanc										
AMF		**	**	**	**	**	**	**	**	**
DS		ns	*	**	ns	**	**	ns	**	**
AMF×DS		ns	*	ns	ns	**	**	ns	**	ns

Table S7. Effect of arbuscular mycorrhizal (AM) fungus on nitrogen: phosphorus in leaves (leaf N:P), stems (stem N:P) and roots (root N:P) of *C. migao* seedlings measured at different experiment stages. Different letters (a, b, c, d) indicate a significant difference of Tukey's post hoc test (** $p < 0.01$; * $p < 0.05$; ns, not significant) between all treatments. PS: prior stress, SS: subjected to drought stress; REC: rewatered; DS: drought stress; WW: well-watered. CK, non-AMF control; *C.etunicatum*, plants inoculated with *C.etunicatum*; *F.mosseae*, plants inoculated with *F.mosseae*; Co-inoculation, plants inoculated with *F.mosseae* & *C.etunicatum*. Values are expressed as the mean ± SE (n = 6, which are treatment replicates).

water regimes	AMF status	Leaf N:P			Stem N:P			Root N:P		
		PS	SS	REC	PS	SS	REC	PS	SS	REC
DS	CK	208.85±3.9a	199.79±12.57a	241.02±16.92a	127.39±24.43	140.56±13.54a	138.99±16.75	201.02±6.2a	153.35±9.95b	162.80±10.7b
	C.	110.86±5.91c	141.22±12.1bc	177.62±26.74b	20.35±1.06b	21.9±1.02c	18.51±0.91c	56.89±2.55b	57.86±2.78d	56.73±1.44c
	F. mosseae	120.14±3.22c	141.36±12.89b	138.4±15.25bc	21.1±2.45b	25.48±2.08c	19.33±0.55c	60.66±5.02b	67.39±3.66cd	66.36±3.98c
	Co-	165.08±14.39	149.08±6.62bc	292.19±49.72a	30.24±1.58b	38.09±2.1c	17.14±0.63c	64.21±3.23b	65.57±4.43d	65.47±4.44c
WW	CK	213.58±6.91a	203.99±15.83a	177.92±14.44b	111.93±14.38a	104.39±17.38	85.91±8.14b	198.11±18.74	209.72±11.66	225.22±16.55
	C.	110.63±4.03c	123.73±5.04c	124.93±2.02c	19.81±0.59b	20.75±0.46c	21.29±0.53bc	56.85±0.75b	57.02±0.8d	58.14±0.5c
	F. mosseae	120.21±2.45c	126.86±6.28c	137.44±6.36c	18.5±1.12b	23.24±0.71c	26.26±0.75bc	82.53±3.31b	83.98±1.76c	80.62±1.39c
	Co-	164.74±13.58	183.37±7.28ab	180.24±8.28bc	32.44±0.8b	30.92±1.21c	31.03±0.98bc	65.88±1.58b	63.77±1.14d	65.71±1.41c
Significanc										
AMF		**	**	**	**	**	**	**	**	**
DS		ns	ns	**	ns	*	**	ns	**	**
AMF×DS		ns	ns	ns	ns	*	**	ns	**	**

Table S8. Effect of arbuscular mycorrhizal (AM) fungus on the soil total concentration of carbon (soil SOM), nitrogen (soil TN) and phosphorus (soil TP) measured at different experiment stages. Different letters (a, b, c, d) indicate a significant difference of Tukey's post hoc test (** $p < 0.01$; * $p < 0.05$; ns, not significant) between all treatments. PS: prior stress, SS: subjected to drought stress; REC: rewatered; DS: drought stress; WW: well-watered. CK, non-AMF control; *C.etunicatum*, plants inoculated with *C.etunicatum*; *F.mosseae*, plants inoculated with *F.mosseae*; Co-inoculation, plants inoculated with *F.mosseae* & *C.etunicatum*. Values are expressed as the mean ± SE (n = 6, which are treatment replicates).

water regimes	AMF status	Soil SOM (g/kg)			Soil TN (g/kg)			Soil TP (g/kg)		
		PS	SS	REC	PS	SS	REC	PS	SS	REC
DS	CK	24.44±2.25ab	19.05±0.53e	40.63±2.2a	1.07±0.05ab	1.61±0.09c	1.38±0.09ab	0.51±0.03a	0.49±0.03b	0.46±0.03b
	C. etunicatum	28.6±1.11a	25.89±1.08abc	31.25±1.73b	1.2±0.16ab	1.95±0.17bc	1.62±0.09ab	0.52±0.01a	0.55±0.01a	0.54±0.01ab
	F. mosseae	24.94±0.8ab	21.99±0.72de	22.31±0.87ef	1.1±0.18ab	2.27±0.08ab	1.6±0.12ab	0.52±0.02a	0.53±0.02a	0.54±0.03ab
	Co-	22.73±1.45ab	18.71±0.52e	20.23±0.79f	1.55±0.13a	2.52±0.15a	1.88±0.15a	0.46±0.02b	0.43±0.02b	0.43±0.02b
WW	CK	24.39±1.84ab	23.23±1.1cd	24.67±1.3cde	1.04±0.07ab	0.94±0.09d	1.22±0.23b	0.52±0.01a	0.52±0.01a	0.55±0.02a
	C. etunicatum	28.07±0.48a	28.14±0.5a	29.78±0.8bc	1.28±0.16ab	1.58±0.09c	1.71±0.03ab	0.52±0.01a	0.57±0.01a	0.59±0.01ab
	F. mosseae	27.56±0.22a	27.43±0.22ab	27.4±0.22bcd	1.28±0.14ab	1.52±0.11c	1.69±0.03ab	0.53±0.01a	0.56±0.01a	0.56±0.01ab
	Co-	23.12±0.79ab	24.47±0.92bcd	20.93±0.5f	1.46±0.08ab	1.48±0.06c	1.59±0.08ab	0.44±0.01b	0.46±0.01b	0.47±0.03b
Significance										
AMF		**	**	**	**	**	**	**	**	**
DS		ns	**	**	ns	**	**	ns	**	**
AMF×DS		ns	**	**	ns	**	**	ns	**	**

Table S9. Effect of arbuscular mycorrhizal (AM) fungus on the soil carbon: nitrogen (soil C:N), carbon: phosphorus (soil C:P) and phosphorus: nitrogen (soil N:P) measured at different experiment stages. Different letters (a, b, c, d) indicate a significant difference of Tukey's post hoc test (** p < 0.01; * p< 0.05; ns, not significant) between all treatments. PS: prior stress, SS: subjected to drought stress; REC: rewatered; DS: drought stress; WW: well-watered. CK, non-AMF control; *C.etunicatum*, plants inoculated with *C.etunicatum*; *F.mosseae*, plants inoculated with *F.mosseae*; Co-inoculation, plants inoculated with *F.mosseae* & *C.etunicatum*. Values are expressed as the mean ± SE (n = 6, which are treatment replicates).

water regimes	AMF status	Soil C:N			Soil C:P			Soil N:P		
		PS	SS	REC	PS	SS	REC	PS	SS	REC
DS	CK	22.99±2.11ab	11.97±0.82bc	30.22±3.25a	47.92±3.67b	38.88±0.67b	88.33±2.74a	2.10±0.08bc	3.29±0.11c	3.00±0.14b
	C. etunicatum	25.75±3.84a	13.83±1.74bc	19.48±1.59bc	55.00±2.22a	47.07±1.58ab	57.87±2.38b	2.31±0.2b	3.55±0.2c	3.00±0.11b
	F. mosseae	25.86±5.3a	9.75±0.6c	14.22±1.07bcd	47.96±1.39b	41.49±1.07b	41.31±1.77c	2.12±0.25bc	4.28±0.1b	2.96±0.16b
	Co-	14.66±1.5bc	7.51±0.4c	10.96±0.72d	49.41±2.73b	43.51±1.57b	47.05±1.91bc	3.37±0.27a	5.86±0.32a	4.37±0.26a
WW	CK	26.13±1.9a	25.62±3.04a	22.42±3.44ab	46.90±2.42b	44.67±1.28b	44.85±1.16c	2.00±0.09bc	1.81±0.11d	2.22±0.26c
	C. etunicatum	23.09±2.53ab	18.05±0.89b	17.39±0.49bcd	53.98±0.87a	49.37±0.76ab	50.47±0.83bc	2.46±0.21b	2.77±0.12d	2.90±0.06b
	F. mosseae	22.47±2.2ab	18.37±1.18b	16.28±0.34bcd	52.00±0.84a	48.98±0.83ab	48.93±0.51bc	2.42±0.17b	2.71±0.13d	3.02±0.05b
	Co-	15.88±0.41b	16.67±1.14b	13.22±0.38de	52.45±1.65a	53.19±1.01a	44.53±0.84c	3.32±0.16a	3.22±0.12c	3.38±0.13b
Significance										
AMF		**	**	ns	**	**	**	**	**	*
DS		ns	**	ns	ns	**	**	ns	**	*
AMF×DS		ns	**	ns	ns	**	**	ns	**	ns
